# Determinants of anemia among women of childbearing age: analysis of the 2018 Mali demographic and health survey

**DOI:** 10.1186/s13690-023-01023-4

**Published:** 2023-01-19

**Authors:** Ebenezer Kwesi Armah-Ansah

**Affiliations:** 1 Department of Population and Health, University of Cape Coast, Cape Coast, Ghana; 2 Department of Population and Development, National Research University - Higher School of Economics, Moscow, Russia; 3 Population Dynamics Sexual and Reproductive Health Unit, African Population and Health Research Center, Nairobi, Kenya

**Keywords:** Mali, Public health, Nutritional anemia, SSA, Prevalence, Maternal health

## Abstract

**Background:**

Anemia occurs at all stages of life and it is of public health concern as it serves as an indicator of quality nutrition and health of a society. Almost one third of the global prevalence of anemia occur among reproductive aged women and almost 40% of these women reside in sub-Saharan Africa including Mali. This study, therefore, sought to assess the determinants of anemia among women of childbearing age in Mali.

**Methods:**

Secondary data analysis of the 2018 Mali Demographic and health Survey (MDHS). Anemia in women was the outcome variable for the study. Data of 5,048 women aged 15–49 was used for the study. Using binary and multivariate logistic regression models, factors associated with anemia among women were identified. The analysis was conducted using Stata version 14.2 software and adjusted Odds Ratio (aOR) with a 95% Confidence Interval (CI) and *p*-value < 0.05 were used to see the significant association.

**Results:**

The prevalence of anemia among women of childbearing age in Mali is 63.5%. Of these, 4.3% and 24.9% were severely and mildly anemic respectively, and the rest 34.3% were moderately anemic. Women who had secondary education (aOR = 0.70, 95% CI: 0.58–0.84), overweight (aOR = 0.63, 95% CI: 0.50–0.81), exposure to mass media (aOR = 0.90, 95% CI = 0.76–1.49) and women with two births (aOR = 0.86, 95% CI = 0.71–1.05) were less likely to be anemic. Likely, richest wealth quintile (aOR = 0.73, 95% CI = 0.51–1.05), living in urban setting (aOR = 0.85, 95% CI = 0.70–1.03) and women in Kidal Region (aOR = 0.42, 95% CI = 0.27–0.65) were less likely to be anemic. However, pregnant women, women covered by health insurance, women with unimproved drinking water, women in communities with low literacy and low socioeconomic status had higher odds of anemia.

**Conclusion:**

These findings point to the need for community and household level public health sensitization interventions to highlight the pro-anemic factors and mitigating strategies. More especially, women with no education, pregnant women, women covered by health insurance, women from communities with low literacy and socioeconomic status ought to be the focus of such interventions.

## Background

Anemia is estimated to have affected more than two billion people globally as of 2016 [1]. Anemia occurs at all stages of life but is more widespread among women of reproductive age (15–49), pregnant women and young children, hence it has become a public health concern in both developing and developed regions [2–4]. Anemia symbolizes poor health and poor nutrition of a society and has adverse consequences on the socio-economic wellbeing of an individual [4, 5]. Anemia is a nutritional deficiency disorder and target 2 of Global Targets 2025 seeks to reduce anemia by half among women of reproductive age by 2025 [6, 7]. To achieve this target, reliable and effective statistics and monitoring systems at the country level would be instrumental [8].

Almost one third of the global prevalence of anemia occur among reproductive aged women and almost 40% of these women reside in developing regions including sub-Saharan Africa (SSA) [9, 10]. Across developing regions, urban–rural variation in anemia exists with a slightly higher proportion in rural areas (44.3%) than in urban areas (40.2%) [11]. This may be due to varied sociocultural contexts with reference to illiteracy, poverty, extent of awareness, cultural and religious taboos, dietary habits, and differences in parasitic infestation [12].

A non-pregnant woman is considered to be anemic if Hb level is below 12 g/dl and below11 g/dl for pregnant women [4, 13]. In low and middle-income countries (LMICs), anemia can be categorized as nutritional deficiencies, infectious diseases, and genetic hemoglobin disorders [14]. However, the prevailing type of anemia is the nutritional anemia, which occurs as a result of poor dieting contributing to iron, folate and vitamin B12 deficiency in the body [15]. Studies have revealed that iron deficiency is largely common among women of childbearing age due to the increase in demand for iron during pregnancy, breastfeeding, menstruation and reproductive cycle associated nutritional deficiencies [16].

It is important to note that anemia impairs the health and well-being of women of reproductive age and increases the risk of maternal and neonatal deaths [17]. Anemia in women of reproductive age may experience preterm birth, stillbirth, increased susceptibility, low birth weight, loss of productivity, fatigue, breathlessness, dizziness, and headaches [18, 19]. Reproductive aged women worldwide are generally more predisposed to iron deficiency than their male counterparts due to body mass index (BMI), age, sex of household head, occupation, marital status, ever had or terminated pregnancy, parity, household size inter alia [15, 20–22]. Anemia leads to various adverse health conditions among childbearing women, ultimately impacting their reproductive capacities [23]. Anemia has caused more than a quarter of maternal deaths, premature births, low birth weight as well as fetal impairment and infant death between 2000 and 2014 [24–27].

Globally prevalence of anemia has increased from an estimated 25% in 2008 to between 29 and 38% [19, 28]. However, SSA is the highest prone region of anemia marked by notable prevalence in some countries within the sub-region such as Mali [5, 29]. West Africa accounts for the highest prone of anemia in SSA and the world [30]. The prevalence of anemia in Mali is almost two-thirds (63%) among women aged 15–49 and they are reported to be suffering from severe degree of anemia. The Kidal region has the lowest anemia prevalence of 48% while Kayes region has the highest prevalence (78%) in Mali [31]. Micronutrient deficiencies, infections usually from malaria, poor socioeconomic conditions and pregnancies have previously been identified as the major causes of anemia among women in Mali [32].

Previous anemia studies in Mali have focused on weight status and iron deficiency [33] and sociodemographic predictors [32]. Besides, there is limited information on anemia among women of childbearing age in Mali. Most researches conducted in Mali did not focus on women of childbearing age although this subpopulation is vulnerable. The current study assesses the determinants of anemia and its determinants among women of childbearing age in Mali. This, therefore, could help government and other stakeholders to design evidence-based public health decisions and consider the implementation of policies and programs that seek to empower women economically in order to reduce the prevalence of anemia. The study will add to the limited knowledge of anemia among women of childbearing age in Mali.

## Materials and methods

### Study area

The study area for this research was Mali which is officially known as the Republic of Mali. Mali is a landlocked country in West Africa and it is the eighth largest country in Africa with a land area of over 1,240,000 square kilometers. Mali is comparatively low at an average elevation of 343m above sea level with Hombori Tondo, the highest mountain peak, is at 1,15 m. The population of Mali is 19.1 million and about 45% of the residents live within cities. The capital of Mali and the biggest city is Bamako [34]. In 2018, the average household size was 5.8 members and the total fertility rate has witnessed a significant decline from 7.1 children per woman in 1987 to 6.3 children per woman. Overweight and obesity has increased over the last two decades from 9 to 28% [31].

### Data source

**Fig. 1:**
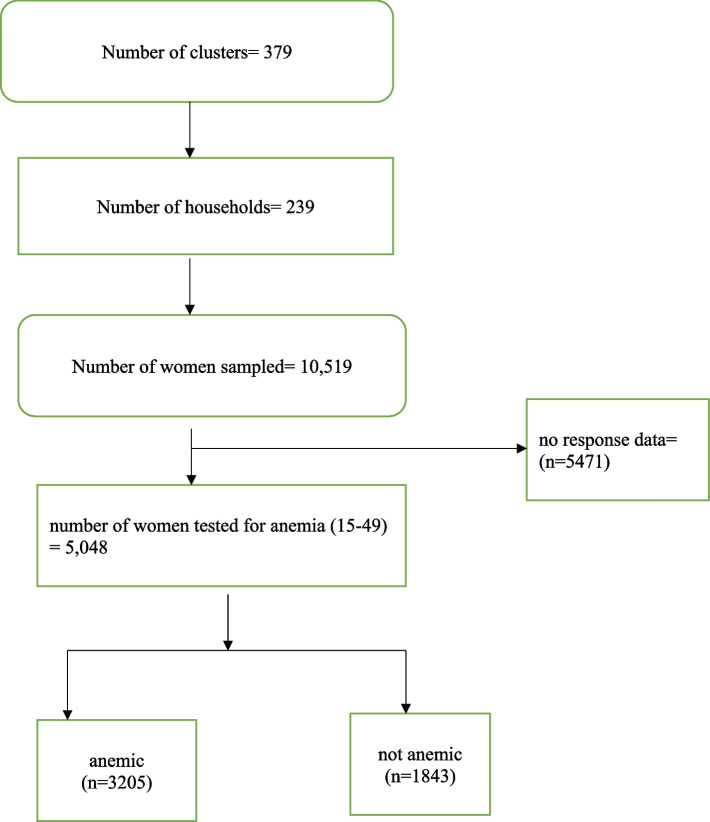
Below is a framework to show the sampling procedure for selecting the study participants

The study utilized data from the 2018 Mali Demographic and Health Survey (MDHS). Specifically, the author used data from the womens’ recode file. The Demographic and Health Survey (DHS) is a nationally representative survey that is conducted in over 85 LMICs globally. The survey focuses on essential maternal and child health markers including anemia in women of reproductive age [35]. The survey employs a two-stage stratified sampling technique and the data captured is nationally representative. The first stage was characterized by the selection of clusters across urban and rural locations from the entire nation. These made-up enumeration areas for the study. The second stage involved the selection of households from the predefined clusters from the previous population survey in Mali. A study provides details of the sampling process [36]. Data collection under the DHS program is done within a year. A total of 5,048 women who had complete data on all the variables of interest were included in this study. The study relied on Strengthening the Reporting of Observational Studies in Epidemiology’ (STROBE) statements. The dataset is freely available for download at: https://dhsprogram.com/data/available-datasets.cfm (Fig. 1).

### Variables of the study

#### Outcome variable

Blood samples were collected from women who voluntarily provided their consent to undertake the hemoglobin test. Those who did not consent were excluded. Following a finger-prick, blood was drawn into a microcuvette for on-site analysis using a battery-operated portable HemoCue analyzer [37]. Blood hemoglobin values were adjusted by altitude among women of childbearing age using this equation:

Hb-adjustment (g/dl) = -0.032 x (altitude × 0.0032808) + 0.022 x (altitude × 0.0032808)^2^ [38].

Based on the CDC formula, the altitude-corrected hemoglobin levels, set under 12 g/dL. In line with WHO definition, anemia was defined as blood hemoglobin level < 12.0 g/dL, which was further categorized as mild (11.0–11.9 g/dL), moderate (8.0–10.9 g/dL), and severe anemia (< 8.0 g/dL). For the purpose of this study, any anemia was defined as blood hemoglobin less than 12.0 g/Dl. That is, the outcome variable was recoded as “Not anemic = 0” (blood hemoglobin more than 12.0 g/dL) whilst “Anemic = 1” (blood hemoglobin less than 12.0 g/dL) [39].

#### Independent variables

Nineteen independent variables were considered in this study based on literature review on the risk of development of anemia among women in LMICs [5, 24]. These are age, educational level, marital status, employment status, Body Mass Index (BMI), parity, subscription to health insurance, exposure to mass media, pregnancy status, terminated pregnancy, region, sex of household head, wealth quintile, source of drinking water, type of toilet facility, number of household member, residence, community literacy level and community socio-economic status. These variables were not determined a priori; instead, based on parsimony, theoretical relevance and practical significance [40–42].

### Definitions of improved and unimproved

Improved drinking-water sources are any water that are either by nature of its construction or through its active intervention is likely to be protected from outside contamination and from faecal matter in particular. On the other hand, unimproved drinking water are not protected from contamination and this includes unprotected well, spring, river dam or lake, stream, pond, rain water and others [43]. In this study, improved drinking water includes pipe water, borehole, protected well, protected spring, tanker trucks, bottled water and bagged water.

Improved type of toilet is the one that hygienically separate human excreta from direct contact with the human body. Therefore, all flushes (pipe, septic, pit latrine), ventilated pit latrine, compositing toilet. However, unimproved types are pit latrine slaps, pit latrine without slaps (open pit), no toilet, bush, bucket toilet, hanging toilet and others [44].

### Statistical analysis

The data were cleaned and analyzed with Stata version 14.2. The analysis was done in two steps. The first step was a bivariate computation of the prevalence and proportions of anemia among women with respect to the independent variables (see Table 1). Chi-square test of independence [X^2^] was used to assess the association between each of the determinants and anemia at a p-value less than 0.05. Variables that showed statistical significance in the bivariate analysis were further moved to the multivariate analysis, which was the second step. The two-level modelling in this study implies that women were nested within clusters. Therefore, clusters were considered as a random effect to cater for the unexplained variability at the community level [1]. Before conducting the Multilevel Logistic Regression analysis, a multi-collinearity test was carried out among all the statistically significant variables to determine if there was evidence of multicollinearity between them. Using the variance inflation factor (VIF), the Multicollinearity test showed that there was no evidence of collinearity among the explanatory variables (Mean VIF = 1.77, Maximum VIF = 3.45, Minimum = 1.03**)**. At the multivariate analysis, a hierarchical binary logistic regression was fitted in four models.

**Table 1:** Distribution of anemia among women in Mali by independent variables (Weighted, *N* = 5048)

Variables	Weighted N (%)	Anemia *n* = 5048 (%)	X^2^	*p*-value
**Age**			**10.6**	**0.101**
15–19	962 (19.1)	630 (65.5)		
20–24	889 (17.6)	539 (60.6)		
25–29	972 (19.3)	610 (628)		
30–34	809 (16.0)	517 (63.9)		
35–39	662 (13.1)	427 (64.5)		
40–44	435 (8.6)	274 (62.9)		
45–49	319 (6.3)	208 (65.2)		
**Education**			**69.4**	0.000^*^
No education	3379 (66.9)	2281 (67.5)		
Primary	660 (13.1)	404 (61.2)		
Secondary +	1009 (20.0)	519 (51.4)		
**Marital status**			**6.1**	**0.191**
Never married	764 (15.2)	460 (60.2)		
Married	4110 (81.4)	2641 (64.3)		
Cohabiting	30 (0.6)	20 (67.8)		
Widowed	67 (1.3)	33 (50.2)		
Divorced	77 (1.5)	50 (64.5)		
**Employment**			**0.5**	**0.497**
Not working	2124 (42.1)	1375 (64.7)		
Working	2924 (57.9)	1829 (62.6)		
**BMI**			**106.8**	0.000^*^
Underweight	481 (9.5)	343 (71.2)		
Normal	3184 (63.1)	2135 (67.1)		
Overweight	1383 (27.4)	726 (52.5)		
**Parity**			**13.4**	0.010^*^
Zero birth	988 (19.6)	631 (63.9)		
One birth	630 (12.5)	380 (60.3)		
Two births	599 (11.9)	355 (59.3)		
Three births	598 (11.8)	384 (64.2)		
Four or more births	2233 (44.2)	1454 (65.2)		
**Covered by Health insurance**			**14.8**	0.000^*^
No	4787 (94.8)	3069 (64.1)		
Yes	261 (5.2)	135 (51.7)		
**Mass media exposure**			**21.1**	0.000^*^
No	942 (18.7)	663 (70.4)		
Yes	4106 (81.3)	2541 (61.9)		
**Pregnancy status**			**10.6**	0.001^*^
No	4466 (88.5)	2802 (62.7)		
Yes	582 (11.5)	402 (69.1)		
**Terminated Pregnancy**			**0.12**	**0.730**
Yes	590 (11.70)	365 (61.7)		
No	4458 (88.3)	2840 (63.7)		
**Region**			**147.4**	0.000^*^
Kayes	751 (14.9)	547 (72.9)		
Koulikoro	945 (18.7)	624 (66.1)		
Sikasso	854 (16.9)	588 (68.8)		
Segou	794 (15.7)	475 (59.8)		
Mopti	528 (10.5)	339 (64.2)		
Toumbouctou	185 (3.7)	122 (66.2)		
Gao	147 (2.9)	103 (70.1)		
Kidal	5 (0.1)	3 (60.0)		
Bamako	839 (16.6)	404 (48.2)		
**Sex of household head**			**0.0**	**0.957**
Male	4229 (83.8)	2687 (63.5)		
Female	819 (16.2)	517 (63.1)		
**Wealth quintile**			**151.3**	0.000^*^
Poorest	867 (17.2)	610 (70.4)		
Poorer	1034 (20.5)	719 (69.6)		
Middle	970 (19.2)	678 (69.9)		
Richer	1063 (21.1)	656 (61.7)		
Richest	1114 (22.1)	541 (48.6)		
**Source of drinking water**			**42.1**	0.000^*^
Unimproved	1446 (28.6)	1026 (71.0)		
Improved	3602 (71.4)	2178 (60.5)		
**Type of toilet facility**			**3.3**	**0.070**
Unimproved	522 (10.3)	361 (69.2)		
Improved	4526 (89.7)	2844 (62.8)		
**Number of household members**				**0.332**
Less than 5	1291 (25.6)	858 (66.5)		
5 +	3757 (74.4)	2346 (62.5)		
**Place of residence**			**113.6**	0.000*
Urban	1283 (25.4)	666 (51.9)		
Rural	3765 (74.6)	2538 (67.4)		
**Community literacy level**			**109.0**	0.000^*^
Low	1831 (36.3)	1317 (72.0)		
Medium	1563 (31.0)	1016 (65.0)		
High	1654 (32.8)	871 (52.7)		
**Community socioeconomic status**			**141.2**	0.000^*^
Low	2959 (2959)	2058 (69.6)		
Moderate	570 (11.3)	378 (66.3)		
High	1519 (30.1)	768 (50.6)		

*p-value less than 0.05 indicates statistical significance*

The first model (Model O) showed the variance in anemia among women of reproductive age attributed to the distribution of the primary sampling units (PSU) in the absence of the explanatory variables. Model (I) took into account only individual variable. In mode II household variables were added to model I and in mode III, which is the complete model, the community level variable factors were added to model II. The STATA command ‘melogit’ was used in fitting these models. Model comparison was done using the log-likelihood ratio (LLR) and the Akaike’s Information Criterion (AIC) tests.

Apart from model O, the results were presented as adjusted odds ratios with their corresponding 95% confidence intervals signifying their level of precision. Sample weight was applied and the survey command (svy) was used to account for the complex sampling design of the survey.

### Ethics approval and consent to participate

This study involved a secondary analysis of data, which did not require further approval since the data is freely available in the public domain. However, the MDHS reports that ethical clearance was obtained from the Ethics Committee of ORC Macro Inc. as well as Ethics Board of the Ministry of Health of Mali. The DHS is guided by the standards for ensuring the protection of respondents’ privacy. Inner City Fund (ICF) International ensured that the survey complied with the U.S. Department of Health and Human Services regulations for the respect of human subjects. The author sought permission from the DHS Program for use of the dataset for this study.

## Results

### Prevalence of anemia in Mali

**Fig. 2:**
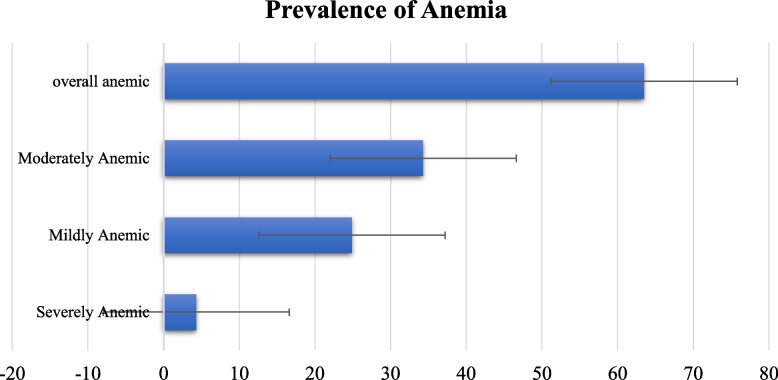
2018 MDHS; Prevalence of anemia among women in Mali

As shown in Fig. 2, the prevalence of anemia among women of childbearing age in Mali is 63.5%. Of these, 4.3% and 24.9% were severely and mildly anemic respectively, and the rest 34.3% were moderately anemic (see Fig. 2).

### Descriptive results

The results showed that 19.3% of the respondents were aged 25–29, 66.9% had no formal education whilst 81.4% were married. More than half (57.9%) of the participants were employed, 63.1% had normal BMI, 44.2% had four or more births. Nearly ninety-five percent of the women were not covered by health insurance policy. With exposure to mass media, 81.3% indicated that they had access to radio, newspaper and television. Majority of the respondents (88.5%) and 18.7% were in the Koulikoro Region. Majority of the respondents (88.3%) had never terminated pregnancy, and 22.1% of the respondents were in the richest wealth group. The findings revealed that 83.8% of the women were in male-headed households. Most of the women (71.4%) had access to an improved source of drinking water and about 90% had access to an improved toilet facility. Seventy-four percent lived with five or more persons and about 75% resided in rural areas. On community literacy level, 36.3% had low literacy and 58.6% had low community socioeconomic status (Table 1).

Women aged 15–19 had the highest prevalence of anemia (65.5%). A significant proportion of women with no formal education (67.5%), those cohabiting (67.8%), those not working (64.7%) and underweight women were anemic (71.2%). Similarly, 65.2% of women with 4 or more births, 64% of women not covered by insurance, and 70.4% of women with no mass media exposure were anemic. Most women who were pregnant (69.1%), living in Kayes (72.9%), and women who had never terminated pregnancy (63.7%) had higher prevalence of anemia. A greater share of women in male-headed homes (63.5%), and 70.4% of those in the poorest quintile category had anemia. Most respondents with unimproved drinking water (71%) and those with unimproved toilet facility (69%) had a higher proportion of anemia. Participants living with less than 5 persons (66.5%), rural dwellers (67.4%), those with low community literacy (72%) and those with low socioeconomic status (69.5%) had the highest prevalence of anemia (Table 1).

### Multivariable hierarchical logistic regression analysis on determinants of anemia among women in Mali

Table 2 shows the hierarchical logistic regression analysis output on determinants of anemia among women in Mali. In model III, having secondary education and above had significant association with a reduced risk of developing anemia among women (aOR = 0.70, 95% CI = 0.58–0.84) compared to no education among women. Overweight women had reduced odds of developing anemia by 37% (aOR = 0.63, 95% CI = 0.50–0.81) compared to women with underweight. Women with two births had lower odds of developing anemia (aOR = 0.86, 95% CI = 0.71–1.05) compared to zero birth. Women who were exposed to mass media had reduced odds of developing anemia by 9% (aOR = 0.90, 95% CI = 0.76–1.06) than women who were not exposed to mass media. Women who were not pregnant at the time of the survey were less likely to have anemia than the pregnant women (aOR = 0.75; 95% CI = 0.62–0.92). Regarding wealth quintile, women who were in the richest wealth quintile had a reduced odds of developing anemia by 27% (aOR = 0.73, 95% CI = 0.51–1.05) while, women in the poorer and middle wealth quintiles were associated with 110% and 106% respectively increase in odds of developing anemia (aOR = 1.10, 95% CI = 0.88–1.37) and (aOR = 1.06, 95% CI = 0.84–1.33) respectively. Having access to improved source of drinking water (0.91, 95% CI = 0.76–1.09) depicted lower odds of anemia. Women in Kidal Region had lower odds of anemia (aOR = 0.42, 95% CI = 0.27–0.65) compared with women in Kayes Region. Women in urban settings had lower odds of anemia (aOR = 0.85, 95% CI = 0.70–1.03) compared with women who reside in rural areas of Mali. Similarly, women in medium community literacy had lower odds of anemia (aOR = 0.84, 95% CI = 0.69–1.01) as compared with those in the low community literacy level. Women in high community socioeconomic status had a reduced risk of developing anemia by 18% (aOR = 0.82, 95% CI = 0.58–1.16). However, women who were covered by health had 1.11 times (aOR = 1.11, 95% CI = 0.83–1.49) higher odds of developing anemia than their counterpart who were not covered by health insurance.

**Table 2:** Multivariable hierarchical logistic regression analysis on determinants of anemia among women in Mali

Variables	Model O	Model I aOR (95% CI)	Model II aOR (95% CI)	Model III aOR (95% CI)
**Fixed effect**				
**Education**				
No education		Ref	Ref	Ref
Primary		0.85 (0.70–1.03)	0.89 (0.73–1.07)	0.86 (0.71–1.04)
Secondary +		0.61^***^ (0.51–0.73)	0.72^***^ (0.60–0.87)	0.70^***^ (0.58–0.84)
**BMI**				
Underweight		Ref	Ref	Ref
Normal		0.90 (0.72–1.12)	0.91 (0.73–1.13)	0.95 (0.76–1.18)
Overweight		0.52^***^ (0.41–0.66)	0.57^***^ (0.45–0.72)	0.63^***^ (0.50–0.81)
**Parity**				
Zero birth		1.05 (0.87–1.25)	1.09 (0.91–1.31)	1.17 (0.97–1.40)
One birth		0.86 (0.71–1.05)	0.89 (0.73–1.09)	0.93 (0.76–1.13)
Two births		0.81* (0.67–0.99)	0.83 (0.68–1.01)	0.86 (0.71–1.05)
Three births		0.93 (0.76–1.14)	0.96 (0.78–1.17)	0.98 (0.80–1.19)
Four or more births		Ref	Ref	Ref
**Covered by Health insurance**				
No		Ref	Ref	Ref
Yes		0.98 (0.73–1.32)	1.12 (0.84–1.51)	1.11 (0.83–1.49)
**Mass media exposure**				
No		Ref	Ref	Ref
Yes		0.82^*^ (0.70–0.96)	0.88 (0.75–1.04)	0.90 (0.76–1.06)
**Pregnancy status**				
Not pregnant		0.74^**^ (0.70–0.96)	0.74^**^ (0.60–0.90)	0.75^**^ (0.62–0.92)
Pregnant		Ref	Ref	Ref
**Wealth**				
Poorest			Ref	Ref
Poorer			1.18 (0.95–1.47)	1.10 (0.88–1.37)
Middle			1.13 (0.90–1.43)	1.06 (0.84–1.33)
Richer			0.91 (0.72–1.15)	0.91 (0.69–1.21)
Richest			0.64^***^ (0.49–0.83)	0.73 (0.51–1.05)
**Source of drinking water**				
Unimproved			Ref	Ref
Improved			0.89 (0.75–1.06)	0.91 (0.76–1.09)
**Region**				
Kayes				Ref
Koulikoro				0.77 (0.57–1.03)
Sikasso				0.76 (0.57–1.02)
Segou				0.55^***^ (0.41–0.74)
Mopti				0.59^**^ (0.42–0.82)
Toumbouctou				0.69^*^ (0.50–0.96)
Gao				0.75 (0.53–1.07)
Kidal				0.42^***^ (0.27–0.65)
Bamako				0.53^***^ (0.37–0.76)
**Place of residence**				
Rural				Ref
Urban				0.85 (0.70–1.03)
**Community literacy level**				
Low				Ref
Medium				0.84 (0.69–1.01)
High				0.96 (0.71–1.30)
**Community socioeconomic status**				
Low				Ref
Moderate				0.87 (0.66–1.14)
High				0.82 (0.58–1.16)
N	5048	5048	5048	5048
Random effect results				
PSU variance (95% CI)	0.58 (0.49–0.67)	0.20 (0.13–0.30)	0.15 (0.09–0.25)	0.11 (0.61–0.21)
ICC	0.093	0.057	0.046	0.033
LR test	X^2^ = 108.33, *p* < 0.001	X^2^ = 42.73, *p* < 0.001	X^2^ = 29.39, *p* < 0.001	X^2^ = 16.24, *p* < 0.001
Wald Chi-square	Ref	217.42	177.27	224.96
Model fitness				
Log-likelihood	-3275.02	-3210.77	-3191.88	-3172.14
AIC	6554.04	6447.54	6419.76	6406.28
BIC	6567.09	6532.38	6537.24	6608.60

Source: 2018 Mali Demographic and health Survey

*PSU* Primary Sampling Unit, *Ref* Reference category, *ICC* Intra-Class Correlation, *LR* Test Likelihood ratio Test, *AIC* Akaike’s Information Criterion; Bayesian information criterion

^*^p < 0.05

^**^p < 0.01

^***^p < 0.001

## Discussion

Anemia among women of reproductive age is fatal because of the rapid physical and mental growth as well as menstruation-associated risks. Anemia is largely preventable and treatable if the determinants at the local or national levels are identified [45]. This study was carried out to assess the prevalence of anemia and its determinants among women of childbearing age in Mali. Anemia is classified as a severe public health problem when the prevalence is more than 39% [46].

The findings from this study indicates that the prevalence of anemia is 63.5%. Of these 4.3% and 24.9% were severely and mildly anemic, and the rest 34.3% were moderately anemic and this a serious public health problem in Mali. This is consistent with the findings of a study in Mali, Nepal, Myanmar and India which revealed that women of childbearing age were anemic [32, 37, 47, 48]. On the contrary, some studies in Rwanda, Brazil and Iran revealed that the prevalence of anemia among women of reproductive age is mild [9, 49, 50]. This could be as a result of dietary patterns, different social and biological susceptibility, geographical and cultural factors [15, 37]. In addition, due to the low socioeconomic status, in Mali, there is insufficient access to meals rich in iron, poor health care access and utilization [15]. This evidently suggests an effective public health related programs and budgetary strategies with appropriate targeting, implementation, monitoring and evaluation in Mali.

From the multivariate logistic analysis, a significant association was found between anemia and education, BMI, parity, covered by health insurance, mass media exposure, pregnancy status, wealth, source of drinking water, region, place of residence, community literacy level and community socioeconomic status.

Consistent with previous studies in Eastern Africa [9, 15, 51, 52] and South Asia [53, 54], this study revealed that women with secondary education and above were less likely to be anemic as compared to women who had no education. This could be that women with higher levels of education may adopt appropriate lifestyle patterns including appropriate health-seeking habits as well as hygiene practices. In addition, these women who have at least secondary education are likely to utilize healthcare facilities, and consume foods that are rich in vitamins and irons which may lead to the reduction of anemia. This suggests a serious policy attention to ensure that at least citizens attain secondary education in Mali.

Similarly, overweight women had a lower prevalence of anemia compared to those with normal BMI. Studies conducted in Mali and Republic of China indicated that women with higher BMI had higher iron consumption and good diet intake as compared to their counterparts with lower BMI [55–57]. On the other hand, a study in Nepal showed a high prevalence of anemia among underweight women [58]. Women suffering from undernourishment are more likely to be deficient in essential micronutrients which may be associated with increased risk of anemia. This suggests a serious policy attention to include micronutrients initiatives as a prioritized program in this particular group in Mali.

In this present study, rich women had a lower risk of developing anemia compared with their counterparts. The finding is in agreement with other studies in Eastern Ethiopia, SSA and Asia [59–61]. In many cases, malnourished women have higher risk of iron deficiency [62], and it is mostly associated with poor socio-economic status [16]. In contrast, a study in Nepal showed that poorer women were less likely to be anemic [63].

The finding from this study is in line with previous studies [13, 37, 64–66] suggesting that women who live in urban setting are less likely to be anemic. Higher anemia prevalence among rural dwellers has been attributed to disparities in health service provision and access, inadequate information, disease risk, fertility preferences and genetic conditions such as sickle cell anemia [21]. However, other studies in SSA [67], and Asia [21, 68] revealed that urban dwellers had a higher risk of developing anemia.

This study has shown that women of reproductive age who were covered by health insurance were more likely to be anemic as compared to those not covered by health insurance. This is parallel with studies in Ghana [69, 70]. The finding from this study may imply that health insurance coverage in Mali is not a protective factor against anemia among women of reproductive. Therefore, the Government needs to cover cases of anemia under health insurance coverage.

Women who reside in Kidal Region had a lower likelihood of being anemic compared with women in Kayes Region. The regional disparity can be attributed to food consumption preferences, the occurrence of communicable diseases and the differences in the availability of healthcare facilities.

There was a significant association between being anemic and parity. Women of reproductive age who had two births were less likely to be anemic than those with four or more children. This may be due to the fact that the more women give birth, the more they are exposed to blood loss leading to low haemoglobin levels in the blood coupled with low-quality meals, parasites. Therefore, there is the need to place emphasis on family planning education and usage [45, 71]. In contrast, a study conducted in Ethiopia reported no statically significant association between anemia and parity [9].

Finding from this study shows that there is a significant relationship between anemia and exposure to mass media. Women of reproductive age in Mali who were exposed to mass media were less likely to be anemic as compared to their counterparts without exposure to mass media. This finding corroborates with some previous studies that have been conducted in Eastern Africa [72, 73].

The study showed pregnancy increased the odds of anemia in women. This is finding is consistent with other studies in Eastern Africa [15]. Studies have revealed that during pregnancy, nutritional deficiencies, bacterial and parasitic infections and genetic disorders could lead to anemia [15, 74]. Also, pregnant women have an increase demand for iron to sustain the baby’s growth. The WHO has approved prenatal use of iron supplements in LMICs to reduce the prevalence of anemia among pregnant women [74]. Therefore, it is essential to provide pregnant women with zinc, iron and folic acid supplement.

Women with improved source of drinking water were less likely to become anemic as compared to those with unimproved source of water. In this current study, the association was significant and this is congruent with a previous study in Nepal [36], Eastern Africa [15, 75] and SSA [75]. The reason could be that women with unimproved source of drinking water are exposed to water borne diseases and this could increase their risk of anemia [76].

Anemia was high (72.0%) among women of childbearing age in Mali who are resident in communities with low literacy. Similarly, the prevalence was high among those with low community socio-economic status (69.5%). There was also a significant association between community literacy level, community socio-economic status and anemia. Women in communities with medium literacy were less likely to be anemic as compared to those with low literacy level. Again, women with high community socioeconomic status were less likely to be anemic. The reason for this variation could be that communities with high literacy and socioeconomic status may influence the kind of food women eat in a positive manner. Also, such communities may have improved source of water and sanitation which could positively influence health status of an individual, hence reduce anemia risk of women in such areas.

Extensive studies have established that socioeconomic status is an important determinant of anemia and the findings from this study reinforce the need for government of Mali and other donors to focus on improving the socioeconomic status of women of childbearing. 

## Strengths and limitations

The major strength of this study is the use of a current nationally representative data of the Mali Demographic and Health Survey in determining the prevalence of anemia and its determinants among women of childbearing age in Mali. Despite this strength, it is worthy to note that some of the limitations inherent in this study. The cross-sectional nature of this study does not allow for causality to be inferred from the findings. Parasitic infections and genetic hemoglobin conditions are known to impact anemia prevalence; however, these were not assessed due to data unavailability. Also, the study did not take into consideration dietary intake among women of childbearing age since it was not captured in the dataset.

## Conclusion

The study revealed that education, BMI, parity, mass media, pregnancy status, health insurance coverage, region, wealth, source of drinking water, place of residence, community literacy level and community socioeconomic status are associated with anemia among woman in Mali. These findings point to the need for community and household level public health sensitization interventions to highlight the pro-anemic factors and recommended mitigating strategies. Attention of women could be drawn to the common factors associated with anemia and available structures and agencies in the society from whom they can seek anti-anemic advice. More especially, women with no education, pregnant women, women living in low socioeconomic status and women who live in communities with low literacy ought to be the focus of such interventions. That is, public health interventions and women empowerment through education and economic status would contribute greatly in averting anemia among women of reproductive age. Therefore, the government of Mali needs to include cases of anemia on health insurance coverage. Furthermore, stakeholders including government and non-governmental organizations may need to consider the implementation of policies and programs that seek to empower women economically in order to reduce the prevalence of anemia. Also, authorities need to prioritize hybrid interventions based on the Sustainable Development Goals (SDGs) including use of iron supplement, promotion of rich iron foods and other micronutrients should be targeted towards women in rural areas in Mali.

## Data Availability

Data is available on https://dhsprogram.com/methodology/survey/survey-display-517.cfm

## Abbreviations

WHO: World Health Organization
LMICs: Low-and-middle-income countries
BMI: Body Mass Index
SSA: Sub-Saharan Africa
MDHS: Mali Demographic and Health Survey
aOR: Adjusted odds ratio
CI: Confidence intervals
